# Disordered eating attitude and associated factors among late adolescent girls in Gondar city, northwest Ethiopia: a community-based cross-sectional study

**DOI:** 10.3389/fpubh.2024.1425986

**Published:** 2024-09-04

**Authors:** Betlihem Muche Engdyhu, Kedir Abdela Gonete, Berhanu Mengistu, Netsanet Worku

**Affiliations:** ^1^Department of Human Nutrition, Institute of Public Health, University of Gondar, Gondar, Ethiopia; ^2^Caring Futures Institute, College of Nursing and Health Sciences, Flinders University, Adelaide, SA, Australia

**Keywords:** disordered eating attitude, late adolescent girl, Gondar city, associated factors, northwest Ethiopia

## Abstract

**Background:**

Disordered eating attitudes are characterized by abnormal beliefs, thoughts, and feelings regarding food and weight control. Dieting, intentional weight loss, and weight control affect 41–62% of girls worldwide. However, there is limited information related to disordered eating attitudes and associated factors among late adolescent girls in Ethiopia, including in the study area. Thus, the study aimed to assess disordered eating attitudes and associated factors among late adolescent girls in Gondar city, northwest Ethiopia.

**Methods:**

For this community-based cross-sectional study, which was conducted from 26 June to 26 July 2022, in Gondar city, Ethiopia, 1,188 adolescent girls were included. Multistage stratified sampling followed by a systematic random sampling technique was applied to recruit participants for the study. Data were collected using an interviewer-administered questionnaire containing the Eating Attitudes Test-26 (EAT-26). Anthropometric measurements were also taken. Bivariable and multivariable logistic regressions were employed to identify factors associated with disordered eating attitudes. In the final model, variables with a *p*-value of < 0.05 were considered statistically significant.

**Results:**

A total of 1,158 late adolescent girls (a response rate of 97.5%) participated in the study. The prevalence of disordered eating attitudes among late adolescent girls was 9.7% [95% confidence interval (CI): 7.96, 11.4%]. Having a mother who was unable to read and write [adjusted odds ratio (AOR): 3.88 (95% CI: 1.07, 14.11)], having a mother who could read and write [AOR: 4.31: (95% CI: 1.14, 16.24)], having a father who only attended primary school [AOR: 3.78 (95% CI: 1.33, 10.78)], having severe anxiety [AOR: 3.26 (95% CI: 1.42, 7.49)], and severe usage of social media [AOR: 2.80 (95% CI: 1.22, 6.46)] were factors significantly associated with disordered eating attitudes among late adolescent girls.

**Conclusion:**

This study revealed that disordered eating attitudes among late adolescent girls were relatively high. The educational status of parents, anxiety, and severe usage of social media were positively associated with disordered eating attitudes. Therefore, nutritional education for parents of adolescents who are vulnerable to unhealthy behavior is imperative. The finding also implied the importance of an inclusive strategy to mitigate the emerging problem of targeting vulnerable groups.

## Background

The recent economic and nutrition transitions documented in developing countries have been an entry point for behavioral changes (1). These changes are common across all age groups but are more pronounced among adolescents (2). In the period of adolescence, there is accelerated growth and development (3). There are also psychological and behavioral changes at this time, and adolescents become more self-conscious and critical of their bodies (4). Consequently, a disordered eating attitude (DEA) emerges (5, 6).

Disordered eating attitudes (DEAs) refer to disordered beliefs, thoughts, and feelings regarding food and weight control, which can occur as a part of an eating disorder (7). DEAs have a substantial effect on the eating practices of individuals. For example, individuals with DEAs engage in calorie restriction, dieting, extreme exercise, self-induced vomiting, and binge eating (8–10). They also involve in restrictive dieting or unhealthy weight and shape management (9). Dieting, intentional weight loss, and weight control affect 41–62% of girls worldwide and they have grown to be a significant public health problem (11).

Even though the disorder frequently occurs in developed countries, recent epidemiological data have revealed a rapid spread of the disorder in other parts of the world (12). Regardless of cultural and religious practices, an increasing number of individuals are engaging in unhealthy eating attitudes and behaviors (13). Additionally, it has been observed that female adolescents are disproportionately engaged in unhealthy weight control behaviors compared to their male counterparts (14).

In adolescent girls, DEA is the third most common disease, next to asthma and obesity (15). The prevalence of DEAs among late adolescent girls (15–19 years) varied from 16.2 to 42.7% in high-income countries (16–18), 4 to 48.9% in middle–upper income countries (19–23), and 24.7 to 31.5% in middle–lower income countries (24, 25). A recent study in Ethiopia also highlighted the existence of DEAs among female adolescents, which is in agreement with the study, where 5.98% of adolescent girls had DEAs (26). In addition, studies have documented that the problem is more prominent among female adolescents than among their male counterparts (26, 27).

A variety of sociodemographic factors have been linked to the rising prevalence of DEAs in adolescent girls. To illustrate, age, marital status of parents (unmarried), educational status of parents (low level) (17, 25, 26), peer pressure (28–30), family pressure (28, 29), family income (17, 29), and residency (17, 30) were found to be the predictors of DEAs among adolescent girls. Moreover, anxiety (18, 21), depression (21), body weight perception (overweight) (21, 25, 30, 31), being obese or overweight (19, 25, 30, 31), social media addiction (20, 30), low self-esteem (31, 32), and limited consumption of animal source food (19) were also positively associated with DEAs among adolescent girls.

In general, adolescent girls with DEAs are more likely to become stunted, which can result in low birth weight babies and obstetric complications, thus preserving the progress of malnutrition from generation to generation (33, 34). Adolescent girls with DEAs are also more likely to manifest fatigue, high blood pressure, reduced learning capacity, delayed sexual maturity, lack of concentration, poor school performance, low self-esteem, and sluggish growth (12).

In cognizance of the dire consequences of poor dietary intake during the period of adolescence, different policies, programs, and strategies have been enacted in Ethiopia to promote optimal nutrition (35). However, decision-makers have placed little focus on behavioral factors that affect optimal nutrition during the adolescent period. In light of this, devising and placing a policy that strives to ameliorate healthy food choices is of paramount importance for the less-empowered groups of the population. Given the dearth of empirical evidence on this issue, the current study hypothesized that the magnitude of DEAs among late adolescent girls (15–19 years) is on the rise, even in resource-limited areas, which owes much to social media use, anxiety, depression, and unhealthy food choices.

Thus, the objectives of this study were 2-fold. The first objective was to estimate the prevalence of DEAs among late adolescent females in Gondar city, Ethiopia, and the second objective was to identify factors associated with DEAs.

## Methods

### Study setting and period

A community-based cross-sectional study was conducted between 26 June and 26 July 2022 in Gondar city. Gondar city is one of the few historical and ancient cities in Ethiopia. It is located ~750 km from Addis Ababa, the capital city of Ethiopia. Based on the Central Statistical Agency (CSA) 2007 report, the estimated population size of the city during the study period was 454,045; out of which, 47,276 were late adolescent girls. Administratively, there were six sub-cities and 37 lowest administrative units (kebeles) in the city. The city had one comprehensive and specialized referral hospital, two general hospitals, and eight health centers.

### Participants and sampling technique

All late adolescent girls who resided in Gondar city for at least 6 months were included in the study. The sample size was estimated using a single population proportion formula by considering 5.98% (26) of the proportion of DEAs among late adolescent girls at a 95% confidence interval (CI) with a 2% margin of error. Finally, after considering 10% contingency and a design effect of 2, 1,188 late adolescent girls were recruited for this study. Adolescent girls who were unable to respond due to illness were excluded.

A multistage sampling technique was used. First, two sub-cities were selected randomly using a lottery method from the six sub-cities in the city. Then, the final sample size was proportionated to the kebeles of the selected sub-cities. Finally, using a systematic random sampling technique, every 12th (*k* ≈ 14,200/1,188) household was employed. When there was more than one adolescent girl in a single household, the participant was randomly selected using the lottery method.

### Data collection

A pretested interviewer-administered questionnaire was used. The data were collected by six trained B.Sc. nurses, and they were supervised by a public health officer. The first section of the questionnaire comprised sociodemographic variables (age, educational status, parents' occupation, family size, living status, and wealth status). Late adolescent girls were defined as those aged between 15 and 19 years. The wealth status was computed using principal component analysis (PCA), using 20 variables that were used to assess housing quality: roofing, walls, and floor material; toilet ownership; and the ownership of durable household tools: chair, table, sofa, mattress, mobile telephone, non-mobile telephone, television, radio, refrigerator, electric stove, bicycle, horse cart, Bajaj (a three-wheel vehicle), and car. These house facilities and durable goods and items were regarded as modern goods that reflected wealth status in urban areas. The responses were dichotomized, with zero indicating non-ownership of the item and one indicating ownership. Finally, they were categorized into poor, medium, and rich.

The second section of the questionnaire contained the Eating Attitudes Test-26 (EAT-26). The EAT has 26 items, each with six responses ranging from always to never. A score of 3 was given for always, 2 for very often, 1 for often, and 0 for occasionally, sometimes, and never. The scores were computed after summing up each response, which ranged from 0 to 78. Finally, adolescent girls who scored 20 and above were considered to have DEAs (26). The reliability of the tool was assessed using a Cronbach's alpha value of 0.70.

Social media usage was measured using the Internet Addiction Test (IAT). The IAT comprised 20-item questionnaires, with each item rated on a 6-point Likert scale (ranging from 0 to 5). The total score was computed; those with a score ranging from 0 to 30 were considered normal users, those with a score between 31 and 49 were considered mild social media users, those with a score between 50 and 79 were considered moderate social media users, and those with a score between 80 and 100 were considered severe social media users (36). The internal consistency (Cronbach's alpha) of the tool (IAT) in this study was 0.96.

The nine-item Patient Health Questionnaire (PHQ-9) was used to define depression among adolescent girls. The questionnaire had nine items with four responses (“not at all-0,” “several days-1,” “more than half of the days-2,” and “nearly every day-3”) for depressive symptoms in the past 2 weeks. The scores ranged from 0 to 27. Then, the scores were categorized into no depression (0–4), mild depression (5–9), moderate depression (10–14), moderately severe depression (15–19), and severe depression (20–27) (37–39). The item showed good internal consistency (Cronbach's alpha, 0.86).

Anxiety was measured using the seven-item General Anxiety Disorder-7 (GAD-7) questionnaire for adolescents. It assessed symptoms related to anxiety in the period of 2 weeks preceding the study. The questionnaire provided the following response options: “not at all-0,” “several days-1,” “over half the days-2,” and “nearly every day-3.” The scores varied between 0 and 21, with the following categories: minimal anxiety (0–4), mild anxiety (5–9), moderate anxiety (10–14), and severe anxiety (15–21) (40). The internal consistency (Cronbach's alpha) of the questionnaire in this study was 0.81.

Body weight perception was defined as the self-evaluation of one's weight as underweight, normal weight, overweight, and obese irrespective of the actual body weight (41).

The weight and height of adolescent girls were measured in accordance with the World Health Organization (WHO) guidelines. A calibrated weight scale was used to measure the weight to the nearest 100 g, and the height was measured to the nearest centimeter with the participant standing in the Frankfurt plane using a stadiometer. During the measurements, the participants did not wear heavy garments or footwears. The weight measuring scale was calibrated to zero from time to time. The AnthroPlus software was used to compute the body mass index (BMI) for the age of adolescent girls. In accordance with the WHO 2007 growth reference, the adolescents were classified as overweight (*Z*-score > +1 SD), normal weight (*Z*-score ≥ −2 SD and +1 SD), and thin (thin < −2 SD) (42).

### Analysis

The data were checked for completeness and clarity. The data were then entered into EpiData 4.6, and statistical analysis was conducted using the Statistical Package for Social Sciences (SPSS) version 25. Descriptive statistics (frequency, percentage, median, and interquartile range) were used to summarize the data, and, figuratively, the findings were presented using tables. Multicollinearity between independent variables was checked using the variance inflation factor (VIF), and multicollinearity was not found (VIF < 10). Binary logistic regression was used to identify the independent predictors of DEAs. Variables with a *p*-value of < 0.2 in the bivariable logistic regression were considered for the final model. The strength of the association was assessed using a crude odds ratio (COR) and adjusted odds ratio (AOR) at 95% CI in the bivariable and multivariable models, respectively. In the final model, a *p*-value of < 0.05 was used to declare significance. The Hosmer–Lemeshow goodness-of-fit test was conducted to check the ability of the model to fit the data (*p* = 0.19).

## Results

### Sociodemographic and economic characteristics of respondents

In this study, 1,158 adolescent girls consented to participate, making the response rate 97.5%. The median age of adolescent girls was 17 ± 3 inter quantile range (IQR), and half (50.8%) of them were in the age group between 17 and 19 years. Almost half (47.3%) of them had completed their secondary school education. The majority (84.6%) had ≥5 family members. More than one-third (35.1%) were from rich households. Regarding the maternal characteristics of the study participants, the majority of them (83.7%) were married and more than one-third (36.4%) had completed their secondary school education (Table 1).

**Table 1:** Sociodemographic and economic characteristics of late adolescent girls in Gondar city, northwest Ethiopia, 2022 (*n* = 1,158).

**Variables**	**Variable category**	**Frequency**	**Percent**
Age in years	15–16	570	49.2
	17–19	588	50.8
Religion	Orthodox	905	78.2
	Muslim	184	15.9
	Protestant	27	2.3
	Jewish	42	3.6
Adolescent girls' educational status	Unable to read and write	54	4.7
	Can read and write	53	4.6
	Primary school (1–8)	448	38.7
	Secondary school (9–12)	548	47.3
	College and above (Higher)	55	4.7
Living status	With my parents	895	77.3
	Outside	263	22.7
Mother's marital status	Single	26	2.2
	Married	969	83.8
	Divorced	55	4.7
	Widowed	49	4.2
	Separated	18	1.6
	Not alive	41	3.5
Mother's educational status	Unable to read and write	118	10.2
	Can read and write	97	8.4
	Primary school (1–8)	291	25.2
	Secondary school (9–12)	424	36.6
	College and above (Higher)	187	16.1
Mother's occupation	Government employee	188	16.2
	NGO	7	0.6
	Merchant	120	10.4
	Daily laborer	50	4.3
	Student	12	1.0
	Housewife	721	62.4
	Job seeker	19	1.6
Father's educational status	Unable to read and write	116	10
	Can read and write	99	8.5
	Primary school (1–8)	238	20.6
	Secondary school (9–12)	400	34.5
	College and above (Higher)	243	21.0
	Not alive	62	5.4
Father's occupation	Government employee	368	31.7
	NGO	22	1.9
	Merchant	296	25.6
	Daily laborer	45	3.9
	Farmer	154	13.3
	Unemployed	59	5.1
	Drivers	28	2.4
	Carpenter	91	7.9
	Housekeeper	33	2.8
Family size	< 5	178	15.4
	≥5	980	84.6
Wealth index	Poor	381	32.9
	Medium	371	32
	Rich	406	35.1

### Psychosocial and behavioral characteristics of respondents

In this study, two-fifths (41.3%) and almost two-thirds (28.2%) of adolescent girls had developed mild depression and severe anxiety, respectively. Furthermore, almost half (49%) of the girls perceived that their weight was normal (Table 2).

**Table 2:** Psychosocial and behavioral characteristics of late adolescent girls, Gondar city, northwest Ethiopia, 2022 (*n* = 1,158).

**Variables**	**Variable category**	**Frequency**	**Percent**
Depression	No depression	317	27.3
	Mild depression	478	41.3
	Moderate depression	208	18
	Moderate severe	67	5.8
	Severe depression	88	7.6
Anxiety	Minimal anxiety	320	27.6
	Mild anxiety	241	20.8
	Moderate anxiety	271	23.4
	Severe anxiety	326	28.2
Body weight perception	Under weight	272	23.5
	Normal weight	567	49
	Overweight	300	25.9
	Obese	19	1.6

### Social media and nutritional status

Of the total, just over half (53.9%) of adolescent girls used social media, with less than one-fifth (14.2%) being normal social media users. In addition, based on the anthropometric measurements, almost three-quarters (71.2%) were normal weight (Table 3).

**Table 3:** Social media and nutritional status of late adolescent girls, Gondar city, northwest Ethiopia, 2022 (*n* = 1,158).

**Variables**	**Variable category**	**Frequency**	**Percent**
Social media use	Yes	624	53.9
	No	534	46.1
Social media	Normal social media	164	14.2
	Mild social media	118	10.2
	Moderate social media	121	10.4
	Severe social media	131	11.3
BMI for age	Thin	102	8.8
	Normal weight	824	71.2
	Overweight	232	20

### Prevalence of disordered eating attitudes

In this study, the overall prevalence of DEAs among late adolescent girls was 9.7% (95% CI: 7.96, 11.4%). Moreover, the study revealed that almost one-tenth (9.6%) of adolescent girls always felt that others would prefer it if they ate more. However, only a small proportion (0.5%) of adolescent girls always had the urge to vomit after meals. A small percentage (8.1%) of the girls were always preoccupied with the thought of having fat on their bodies (Table 4).

**Table 4:** EAT-26 score among late adolescent girls, Gondar city, northwest Ethiopia, 2022 (*n* = 1,158).

**Variables**	**Always (%)**	**Very often (%)**	**Often (%)**	**Sometimes, rarely, and never (%)**
Find myself preoccupied with food	31 (2.7%)	56 (4.8)	80 (6.9)	991 (85.6)
Have binge eaten where I feel that I am not able to stop	9 (0.8)	5 (0.4)	1 (0.1)	1,143 (98.7)
Have vomited after I have eaten	5 (0.4)	6 (0.5)3	1 (0.1)	1,146 (99)
Feel that food controls my life	25 (2.2)	71 (6.1)	92 (7.9)	970 (83.8)
Give too much time and thought to food	31 (2.7)	84 (7.3)	78 (6.7)	965 (83.3)
Have the urge to vomit after a meal	6 (0.5)	1 (0.1)	1 (0.1)	1,150 (99.3)
Avoid eating when I am hungry	13 (1.1)	34 (2.9)	37 (3.1)	1,074 (92.7)
Cut my food into pieces	57 (4.9)	75 (6.5)	51 (4.4)	975 (84.2)
Feel that others would prefer it if I ate more	111 (9.6)	99 (8.5)	225 (19.4)	723 (62.4)
I am preoccupied with the thought of having fat on my body	96 (8.1)	92 (7.8)	111 (9.4)	859 (72.5)
Take longer than others to eat my meal	96 (8.3)	92 (7.9)	111 (9.6)	859 (74.2)
Display self-control around food	19 (1.6)	66 (5.7)	48 (4.1)	1,025 (88.5)
Feel that others pressure me to eat	101 (8.7)	108 (9.3)	218 (18.8)	731 (63.1)
Terrified of being overweight	98 (8.5)	121 (10.4)	135 (11.7)	804 (69.4)
Aware of the calorie content of the food eaten	8 (0.7)	5 (0.4)	7 (0.6)	1,138 (98.3)
Particularly avoid food with a high carbohydrate content	8 (0.7)	16 (1.4)	7 (0.6)	1,127 (97.3)
Feel extremely guilty after eating	18 (1.6)	67 (5.8)	52 (4.5)	1,021 (88.2)
I am extremely preoccupied with the desire to be thinner	56 (4.8)	54 (4.7)	61 (5.3)	987 (85.2)
Think of burning up calories when I exercise	30 (2.6)	78 (6.7)	78 (6.7)	972 (83.9)
Other people think I am too thin	86 (7.4)	60 (5.2)	95 (8.2)	917 (79.2)
Avoid food with sugar in them	13 (1.1)	37 (3.2)	56 (4.8)	1,052 (90.8)
Eat diet food	9 (0.8)	4 (0.3)	11 (0.9)	1,134 (97.9)
Feel uncomfortable after eating sweets	21 (1.8)	35 (3.0)	74 (6.4)	1,028 (88.8)
Engage in dieting behavior	14 (1.2)	80 (6.9)	61 (5.3)	1,003 (86.6)
Like my stomach to be empty	129 (11.1)	210 (18.1)	186 (16.1)	633 (54.7)
Enjoy trying new rich food	163 (14.1)	520 (44.9)	135 (11.7)	340 (29.4)

### Factors associated with disordered eating attitudes among late adolescent girls

In the bivariable logistic regression analysis, the age of adolescent girls, the living status in the family, the mother's educational status, the father's educational status, household wealth index, anxiety, depression, social media use, family size, and nutritional status showed a significant association with DEAs at a *p*-value of <0.2. Subsequently, these variables were fitted to the multivariable logistic regression analysis to control for potential confounders. Accordingly, it was noted that having a mother who was unable to read and write, having a mother who could read and write, having a father who had attended primary school, having severe anxiety, and having severe social media use were independently associated with DEAs (*p* < 0.05 (Table 5). In addition, the fitness of the model was assured using the Hosmer–Lemeshow goodness-of-fit test (*p-*value = 0.19) and adjusted R square (*p-*value = 0.28).

**Table 5:** Bivariable and multivariable logistic regression analysis of the factors associated with DEAs among late adolescent girls in Gondar city, northwest Ethiopia, 2022 (*n* = 1,158).

**Variables**	**Variable category**	**DEAs**		**Bivariable analysis**			**Multivariable analysis**		
				**Unadjusted coefficient**		**COR (95% CI)**	**Adjusted coefficient**		**AOR (95% CI)**
		**Yes (EAT** ≥**20)**	**No (EAT**<**20)**	β	**SE**		β	**SE**	
Age (years)	15–16	36	534			1			1
	17–19	76	512	0.79	0.21	2.20 (1.45, 3.33)^*^	−0.18	0.35	0.83 (0.42, 1.65)
Living status	With my parents	75	820			1			1
	Outside	37	226	0.58	0.21	1.79 (1.18, 2.72)^*^	−0.37	0.43	0.69 (0.29, 1.60)
Mother's education status	Unable to read and write	20	98	0.71	0.35	2.04 (1.02, 4.08)^*^	1.36	0.66	3.88 (1.07, 14.11)
	Can read and write	22	75	1.02	0.35	2.93 (1.47, 5.84)^*^	1.46	0.68	4.31 (1.14, 16.24)^*^
	Primary school (1–8)	22	269	−0.20	0.34	0.82 (0.42, 1.58)	−0.58	0.56	0.56 (0.18, 1.68)
	Secondary school (9–12)	21	403	−0.65	0.34	0.52 (0.27, 1.01)	−1.03	0.55	0.36 (0.12, 1.04)
	College and above	17	170			1			1
Father's education status	Unable to read and write	18	98	1.35	0.4	3.87 (1.76, 8.50)	0.4	0.7	1.49 (0.38, 5.96)
	Can read and write	18	81	1.54	0.4	4.69 (2.12, 10.34)	0.49	0.68	1.63 (0.43, 6.24)
	Primary school (1–8)	31	207	1.15	0.36	3.16 (1.55, 6.44)	1.33	0.53	3.78 (1.33, 10.78)^*^
	Secondary school (9–12)	22	378	0.2	0.38	1.23 (0.58, 2.58)	0.77	0.53	2.16 (0.77, 6.11)
	College and above	11	232			1			1
Family size	<5	11	167			1			1
	≥5	101	879	0.56	0.33	1.74 (0.92, 3.32)	1.06	0.66	2.88 (0.79, 10.46)
Wealth status	Poor	42	339	0.34	0.24	1.40 (0.87, 2.26)	0.06	0.43	1.06 (0.46, 2.45)
	Medium	37	334	0.23	0.25	1.25 (0.77, 2.05)	0.54	0.37	1.72 (0.83, 3.57)
	Rich	33	373			1			1
Depression	No depression	23	294			1			1
	Mild depression	45	433	0.28	0.27	1.33 (0.79, 2.24)	−0.39	0.41	0.68 (0.30, 1.52)
	Moderate depression	27	181	0.64	0.29	1.91 (1.06, 3.43)	0.51	0.48	1.66 (0.65, 4.28)
	Moderate severe	8	59	0.55	0.44	1.73 (0.74, 4.06)	0.73	0.66	2.08 (0.58, 7.55)
	Severe depression	9	79	0.38	0.41	1.46 (0.65, 3.27)	0.9	0.66	2.46 (0.67, 9.05)
Anxiety	Minimal anxiety	19	301			1			1
	Mild anxiety	20	221	0.36	0.33	1.43 (0.75, 2.75)	0.28	0.51	1.33 (0.49, 3.60)
	Moderate anxiety	21	250	0.29	0.33	1.33 (0.70, 2.53)	0.52	0.49	1.68 (0.64, 4.41)
	Severe anxiety	52	274	1.1	0.28	3.01 (1.73, 5.21)^*^	1.18	0.42	3.26 (1.42, 7.49)^*^
Social media use	Normal social media	15	149			1			1
	Mild social media	15	103	0.37	0.39	1.45 (0.68, 3.09)	0.15	0.48	1.162 (0.46, 2.95)
	Moderate social media	12	109	0.09	0.41	1.09 (0.49, 2.43)	0.29	0.48	1.35 (0.53, 3.42)
	Severe social media	25	106	0.85	0.35	2.34 (1.18, 4.66)^*^	1.03	0.43	2.80 (1.22, 6.46)^*^
BMI for age	Thin	6	96			1			1
	Normal weight	58	766	0.19	0.44	1.21 (0.51, 2.88)	−0.44	0.73	0.64 (0.15, 2.68)
	Overweight	48	184	1.43	0.45	4.17 (1.72, 10.10)^*^	0.77	0.75	2.16 (0.49, 9.39)

^*^p-value < 0.05.

When compared to adolescent girls whose mothers had attended college and above, the odds of developing DEAs were 3.88 [AOR: 3.88 (95% CI: 1.07, 14.11)] times higher for adolescent girls whose mothers were unable to read and write and 4.31 [AOR: 4.31: (95% CI: 1.14, 16.24)] times higher for adolescent girls whose mothers could read and write. When comparing adolescent girls whose fathers had only primary school education to adolescent girls whose fathers had higher education, the odds of having DEAs was 3.78 [AOR: 3.78 (95% CI: 1.33, 10.78)] times higher for the former group.

Furthermore, adolescent girls with severe anxiety had 3.26 [AOR: 3.26 (95% CI: 1.42, 7.49)] times higher odds of developing DEAs than those with minimal anxiety. When comparing late adolescent girls with normal social media usage to those with severe social media use, the odds of having DEAs was 2.8 [AOR: 2.80 (95% CI: 1.22–6.46] times higher among the latter group (Table 5).

## Discussion

This study was undertaken to estimate the prevalence and associated factors of disordered eating attitudes (DEAs) among late adolescent girls in Gondar city. The study indicated that the prevalence of DEAs among late adolescent girls was 9.7% (95% CI: 7.96, 11.4%). This finding was in line with a previous study conducted in Singapore (10.5%) (43). However, the prevalence found in this study was higher than in a study conducted in Addis Ababa, Ethiopia (5.98%) (26). The difference in the study period might explain the discrepancy. In recent years, along with the increased availability of smartphones, social media users in Ethiopia have been increasing on a daily basis, which has become an entry point to Western lifestyles (44, 45). Unregulated usage of these social media outlets in Ethiopia could lead to body dissatisfaction and anxiety, which is associated with an increased rate of DEAs (18). Moreover, this study also revealed a higher proportion of DEAs among adolescents when compared to a report from China (4%) (17). The controlled use of social media among adolescents in China might explain this decreased rate (46).

On the contrary, the prevalence of DEAs in the current study was lower than that reported in other African countries, such as Nigeria (13.1%) and Egypt (35.5%) (24, 25). The observed discrepancy could be explained by the Western culture that has been imposed upon these African countries due to the strong ties that they have established over the centuries (47, 48). Furthermore, this finding was lower than the findings from countries in the Middle East, such as Iran (24.7%) (21), Saudi Arabia (25.47%) (19), and Jordan (25.47%) (31). Rapid economic changes that resulted in sociocultural changes in Middle Eastern countries might explain the difference (49). Moreover, this finding was lower than the findings reported in European countries, such as Bulgaria (22.5%) (10), Greece (17.8%) (18), and Spain (29.87%) (16). The possible reasons for this difference could be sociocultural differences, such as considering thinness as a standard of beauty in industrial society. In addition, an increased consumption of processed foods in these countries could result in rapid weight gain and obesity, which later predisposes adolescents to depression and body image dissatisfaction (50).

This study revealed that mothers' educational status has a positive association with DEAs among late adolescent girls. The odds of having DEAs were higher among late adolescent girls whose mothers were unable to read and write and the girls whose mothers could only read and write when compared to late adolescent girls whose mothers had a higher educational status (college and above). This was comparable with a report from Addis Ababa (26). This might be due to the fact that a high level of parental education would create a healthy lifestyle. Clearly, educated mothers could provide advice to their daughters on the importance of following healthy nutritional practices (51, 52).

Similarly, the odds of developing DEAs were higher among late adolescent girls whose fathers had primary school education compared to the adolescents whose fathers had a higher educational status (college and above). This result was in line with a previous study conducted in Singapore (43). The possible reason is that increased literacy raises people's awareness regarding various preventive actions for a healthy lifestyle (52).

The odds of developing disordered eating attitudes among adolescent girls who developed severe anxiety were higher than adolescent girls who developed minimal anxiety. This was consistent with earlier findings reported in Iran and Greece (18, 21). Increased anxiety levels may lead adolescents to maladaptive ways of managing their stress and emotions, through disordered eating. When one eats more to cope with a stressful situation, then one may exercise more or eat less the next day to offset the caloric over-consumption, which in turn results in DEAs (53).

Furthermore, the odds of developing DEAs among adolescent girls with severe social media usage were higher than among adolescent girls whose social media usage was normal. This was comparable with a study from the Philippines and Turkey (54, 55). The possible reason is that repeated exposure to social media could direct individuals' perceptions and views toward body appearance in a different direction. For example, in different social media outlets, a thin woman is often portrayed as an ideal woman, which has encouraged girls to lose weight and engage in dieting (56).

There are some limitations in the present study. On assessing depression and anxiety over a period of 2 weeks, the participants may have been prone to recall bias. In addition, the cross-sectional nature of the study may have limited the establishment of causal associations between the predictors and disordered eating attitudes. Finally, as male adolescents were not included in this study, the findings of this study should be carefully interpreted for all late adolescents.

## Practical implication

In developing countries, such as Ethiopia, the problem of eating disorders among adolescents is unsolicited in public health research. Due to this reason, identifying the causes of disordered eating to provide an effective solution has been a challenge in the country. In light of this, the current study attempted to provide baseline information for researchers and policymakers to design an inclusive strategy for mitigating this emerging problem.

This study also revealed that social media influence (severe social media use) is an important challenge in disordered eating attitudes. It is, therefore, noteworthy to create an awareness that the body image that is being advertised in different social media outlets is unattainable. Moreover, the findings of this study implied the importance of developing messages that can be used by educators, social media influencers, and parents in raising awareness to limit social media's influence on body image.

## Conclusion

The current study revealed that the rate of disordered eating attitudes among late adolescent girls was relatively high. Having a mother who was unable to read and write, having a mother who could only read and write, having a father with a primary school education, participants with severe anxiety, and a severe social media user were significantly associated with disordered eating attitudes among late adolescent girls in Gondar city. Therefore, nutritional education for the parents of adolescents who are vulnerable to unhealthy behavior is imperative for alleviating the problem. In addition, parental control and legislation that limit social media usage among adolescent girls need to be encouraged.

## Data Availability

The raw data supporting the conclusions of this article will be made available by the authors, without undue reservation.
